# Surface coating preparation of spherical magnetic materials

**DOI:** 10.1039/d2ra00806h

**Published:** 2022-03-30

**Authors:** Zixu Ma, Beibei Yang, Junjiao Yang

**Affiliations:** College of Chemistry, Beijing University of Chemical Technology Beijing 100029 China mazixubuct@163.com yangbeibei8896@163.com; Analysis and Test Center, Beijing University of Chemical Technology Beijing 100029 China yangjj@mail.buct.edu.cn

## Abstract

Magnetic materials are being increasingly used in anti-counterfeiting coatings, but the dark colors of magnetic materials greatly limit their applications. This necessitates the development of light-colored magnetic materials. In this study, the heterogeneous precipitation method was used to deposit a layer of titanium dioxide (TiO_2_) on the surface of magnetic spherical metal particles, followed by the deposition of a layer of Ag by the reduction method, in order to achieve a light color. In the experiment, the particles were initially coated with a few tens of nanometers of TiO_2_ with a strong shading effect, followed by a further coating of Ag of the same thickness with a similar shading performance. Not only did this achieve a lighter color, but there was no reduction in the magnetic properties of the material after the application of the coating. Scanning electron microscopy (SEM), scanning electron microscopy and energy-dispersive spectroscopy (SEM-EDS), X-ray diffractometry (XRD), and other methods were used to study the changes in morphology and composition before and after the magnetic material was coated. A magnetic tester was used to study the changes in magnetic strength before and after the magnetic material was coated.

## Abstract 2

Magnetic materials are being increasingly used in anti-counterfeiting coatings, but the dark colors of magnetic materials greatly limit their applications.

## Graphical abstract

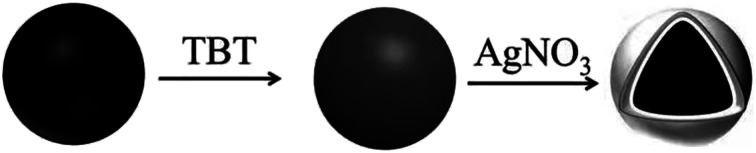

## 1. Introduction

Magnetic materials play an important role in our daily lives, being of critical importance to the field of energy and its applications (*i.e.*, motors, generators, transformers, actuators, *etc.*).^1^ With the advancing research into magnetic materials, many innovative applications are now possible, including magnetic fluids, labeling and classification of biological species, biomedical imaging, site-specific drug delivery, and magnetic cell lysis, particularly by modifying the surface of magnetic material particles.^22^ The surface color can greatly broaden the application range of such particles.

Composite particles with a core–shell structure have important application prospects in catalysis, magnetism, and composite materials science.^2^ The preparation methods of core–shell composite particles include chemical reduction, high-energy ball milling, sol–gel, chemical precipitation, chemical plating, and heterogeneous nucleation methods.^3,4^ The heterogeneous precipitation method has a multitude of benefits: uniform particle distributions, lack of agglomeration, a simple synthesis process, and low cost. Therefore, it has become the most widely used method for coating the surface of particles with titanium dioxide (TiO_2_). TiO_2_ is a photocatalyst that can be used in solar cells, water splitting, pollutant degradation, and other applications.^5,6^ It is used in photocatalysis,^7,8^ self-cleaning coatings,^9^ and hydrogen production.^10,11^ Photocatalytic sensors^12,13^ have a wide range of potential applications. Additionally, TiO_2_ has excellent opacity, whiteness, and brightness and is considered to be the best white pigment currently available; it has good hiding power and strong adhesion, and is an exceptionally white inorganic pigment used in paints^14,15^ and as a matting agent in enamels. Metallic silver (Ag), which is silvery-white, not only has good electrical and thermal conductivity, but also excellent shading properties.^16–18^ The color of magnetic materials, whether these are metal oxides or metal materials, is relatively dark, which greatly limits their applications. The use of light-shielding materials to mask the color of magnetic materials has therefore become a topic of great interest in recent years. Since the shortest wavelength of visible light is approximately 400 nm, it is necessary to coat the surface of the magnetic material with a layer of high-light-shielding material with a thickness of more than 400 nm in order to conceal the color of dark magnetic materials. TiO_2_ coating on the surface of magnetic materials can provide a good light-shielding effect, but requires a very thick coat of TiO_2_, which greatly reduces the magnetic properties of magnetic materials. Furthermore, TiO_2_ has a strong effect on organic matter. Photodegradation can also be problematic;^19^ if a magnetic material coated with TiO_2_ is directly applied to an organic polymer, the organic polymer in contact with the magnetic material will be degraded.^20,21^ This study examines the use of an initial coating of a layer of tens of nanometers of TiO_2_ on the surface of the magnetic material, followed by a layer of tens of nanometers of Ag. Not only does this avoid the degradation of organic matter by TiO_2_, but it also makes the magnetic material well. Thus, the color becomes lighter without affecting the magnetic properties.

## 2. Experimental materials and methods

### 2.1 Materials

Iron–cobalt magnetic material (Fe–Co50); tetrabutyl titanate (TBT; analytical grade, Aladdin reagent); ethyl orthosilicate (analytical grade, Aladdin reagent); polyvinylpyrrolidone (PVP; chemically pure, Beijing Chemical Reagent Company); trimethylhexadecylammonium bromide (CTAB; analytical grade, Aladdin reagent); silver nitrate (AgNO_3_; analytical grade, Aladdin reagent); ammonia water (analytical grade, Fuchen (Tianjin) Chemical Reagent Co., Ltd); and absolute ethanol (analytical pure, Tianjin Fuyu Fine Chemical Co., Ltd) were used.

### 2.2 Instruments

A scanning electron microscope (SEM) (S-4700, Hitachi, Japan); an energy-dispersive spectroscopy (EDS) annex for the scanning electron microscope (EDAX, USA); an X-ray diffractometer (D/max 2500 VB2 PC, Rigaku, Japan); and a 7400 series vibrating sample magnetometer (Lake Shore, USA) were employed.

### 2.3 Preparation method

#### 2.3.1 Preparation of Fe–Co50@TiO_2_

A total of 3 g of Fe–Co50 was dispersed in a solution of 50 mL ethanol and 8 mL deionized water. Following the addition of 2 mL 1% CTAB, the above solution was transferred to a 250 mL three-necked flask, and stirred for 20 min in an ice-water bath. Then, 3 mL TBT was placed in a constant-pressure dropping funnel, which slowly it into the Fe–Co50 solution. After the addition was complete, the solution was stirred for another 2 h in an ice-water bath. Subsequently, the temperature of the reaction mixture was raised to 50 °C, and the solution was allowed to stir and react for 2 h. Then, the temperature was raised to 75 °C and the solution was stirred again for 4 h, at which point the reaction was stopped. The reactants were ultrasonically cleaned with ethanol 3–5 times and dried in a blast drying box at 80 °C to obtain a titanium-dioxide-coated magnetic material (Fe–Co50@TiO_2_).

#### 2.3.2 Configuration of silver ammonia solution

A total of 1 g of AgNO_3_ was dissolved in 10 mL water and stirred. After the dissolution was complete, concentrated aqueous ammonia was added to the AgNO_3_ solution. The addition of aqueous ammonia was stopped at the point when the solution turned from turbid to clear.

#### 2.3.3 Preparation of Fe–Co50@TiO_2_@Ag

**Scheme 1:**
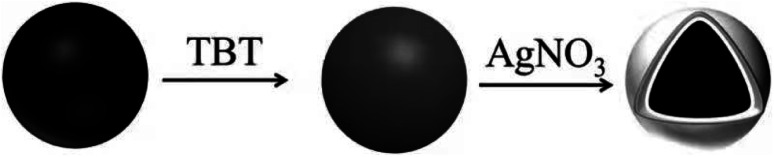
Schematic of the formation of Fe–Co50@TiO_2_@Ag.

A total of 10 g of Fe–Co50@TiO_2_ was placed in a 250 mL three-necked flask. Then, 100 mL of water was added, followed by 5 g glucose and 0.5 g PVP. The solution was stirred for 20 min in a water bath set to 25 °C, and the silver ammonia solution prepared as described in Section 2.3.2 was gradually added dropwise to the reaction mixture. The solution was left to react for 4 h after the addition was complete. The reactant was ultrasonically cleaned 3–5 times with ethanol and dried in a blast drying box at 80 °C to obtain a silver-coated, titanium-dioxide-coated magnetic material (Fe–Co50@TiO_2_@Ag). Scheme 1 illustrates the formation of Fe–Co50@TiO_2_@Ag.

## 3. Results and discussion

### 3.1 TiO_2_ coating amount and feed ratio

TBT undergoes a hydrolysis reaction in the ethanol–water system to generate TiO_2_, which is deposited on the surface of Fe–Co50 magnetic particles. The hydrolysis reaction rate of TBT is known to have a positive correlation with temperature. The ice water bath conditions can decrease the hydrolysis rate and regulate TiO_2_ production. However, the hydrolysis reaction of TBT is relatively fast and non-selective. If it is not induced and controlled, TiO_2_ particles will be produced in the solution, and the Fe–Co50 surface will not be coated. CTAB forms micelles in the ethanol–water system and binds water to the micelles of the *tert*-butyl hydrophilic group. The surface of the Fe–Co50 magnetic particles is a polar surface. After CTAB is added, it forms micelles on, and binds water to, the surface of Fe–Co50, causing the hydrolysis reaction of TBT to proceed only on the surface of Fe–Co50. This achieves better control of the reaction in a heterogeneous phase.

**Fig. 1:**
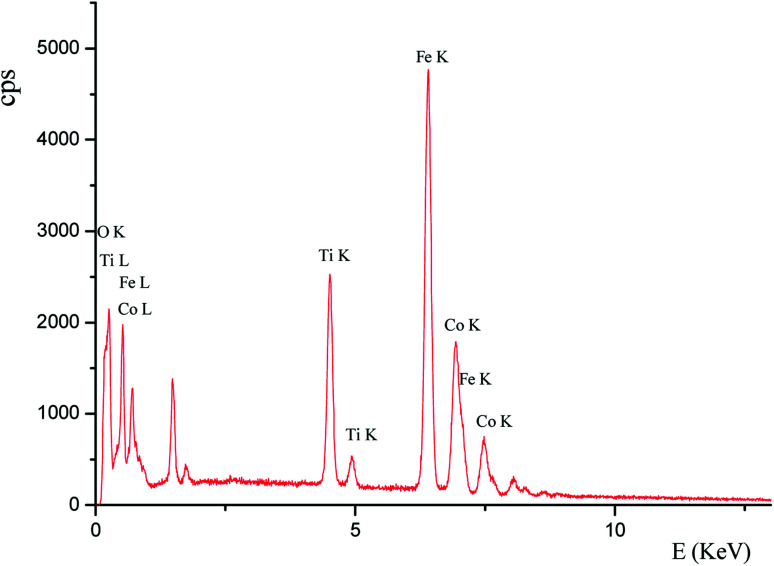
SEM-EDS plot of Fe–Co50@TiO_2_.

**Fig. 2:**
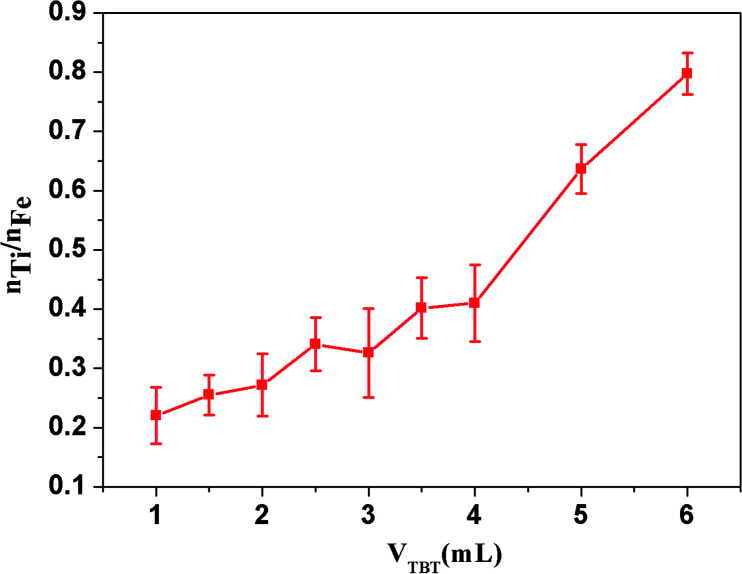
Relationship between the Ti/Fe atomic ratio and TBT dosages.

To shade the magnetic material without affecting its magnetic properties, the relationship between the amount of TBT and the amount of coating on the surface of the magnetic material was studied. Scanning electron microscopy and energy dispersive spectroscopy (SEM-EDS) was used to quantitatively study the coating of TiO_2_ on Fe–Co50 (see Fig. 1). The working conditions of the SEM-EDS used in this experiment were an accelerating voltage of 20 kV and a working distance of 12 cm, unless stated otherwise. SEM-EDS was used to detect the elemental composition of magnetic materials at different dosages. The amount of Ti corresponds to the coating amount of TiO_2_ and that of Fe represents the amount of Fe–Co50 magnetic material. The amount of Fe–Co50 was kept constant and different dosages of TBT were studied. Specifically, the molar ratios of Ti and Fe atoms were compared (see Fig. 2). The atomic ratio of Ti to Fe can quantitatively describe the degree of coating of TiO_2_ on the surface of Fe–Co50. Fig. 2 shows the molar ratio of Ti to Fe on the ordinate, and the abscissa is the volume of TBT under the condition that the mass of both Fe–Co50 samples is 3 g. It can be seen from Fig. 2 that the coating amount of TiO_2_ on the magnetic material increases almost directly proportionally with the dosage of TBT, that is, as the dosage of TBT increases, the coating thickness also gradually increases.

**Fig. 3:**
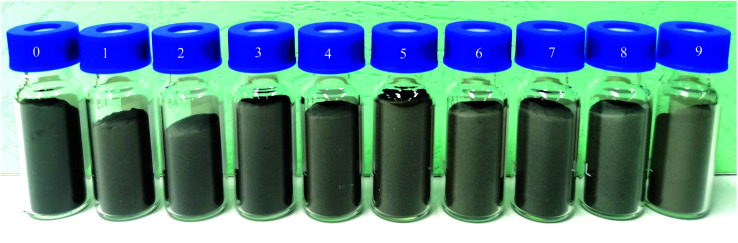
TiO_2_ coating amount and Fe–Co50@TiO_2_ color comparison chart; mass of Fe–Co50 is 3 g, volume of TBT is 0, 1, 1.5, 2, 2.5, 3, 3.5, 4, 5, and 6 mL, respectively.

Samples of the magnetic materials with different coating thicknesses were transferred to vials and photographed. Fig. 3 shows the color change of Fe–Co50@TiO_2_ under different TiO_2_ coating amounts. For 3 g Fe–Co50, when the dosage of TBT is gradually increased from 1 mL to 6 mL, the TiO_2_ coating amount gradually increases, the coating thickness gradually increases, and the color gradually becomes lighter. Increasing the coating amount of TiO_2_ is a gradual process resulting in a change in the apparent color of Fe–Co50, but the color of Fe–Co50 will not fundamentally change by increasing the TiO_2_ coating amount alone. The amount of TBT compared to Fe–Co50 added (*i.e.*, the feed ratio) is approximately *V*_TBT_ : *W*_Fe–Co50_ (1 : 1, mL : g). Under the above feed ratio, the coating thickness is in the range of 20–30 nm (confirmed by SEM as discussed in Section 3.3). Above a TBT dosage of *V*_TBT_ : *W*_Fe–Co50_ = 1 : 1 (mL : g), the color of Fe–Co50@TiO_2_ hardly changes when the coating amount is increased. This has been used as the feed ratio from this point on, unless specified otherwise.

### 3.2 Ag coating amount and feed ratio

The silver ammonia solution of AgNO3 reacts with glucose to produce elemental silver (Ag). The addition of PVP during the reaction can induce Ag deposition on the surface of Fe–Co50@TiO_2_; the role of PVP is to control the growth direction and crystal size of the silver crystals to form a dense coating structure.

**Fig. 4:**
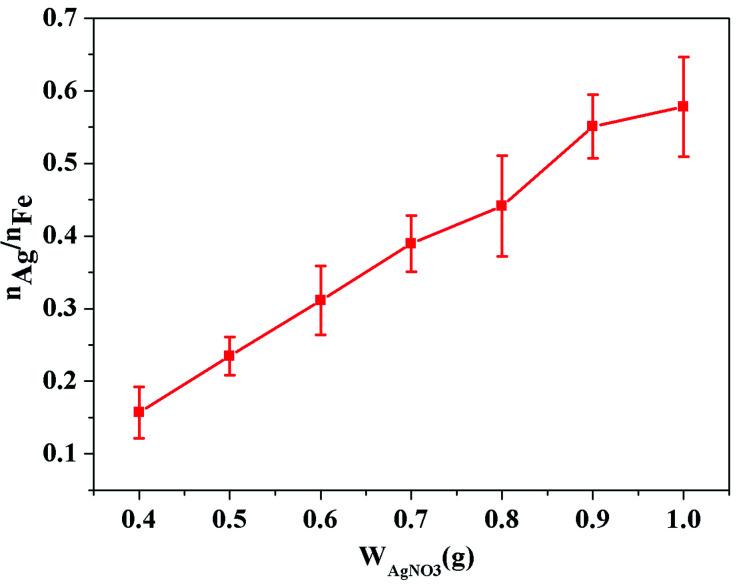
Relationship between Ag/Fe atomic ratio under different AgNO_3_ additions.

From the results discussed in Section 3.1, it is known that above a certain thickness of TiO_2_ coating, increasing the coating amount of TiO_2_ has little effect on the color change. Therefore, Fe–Co50@TiO_2_ was prepared with a feeding ratio of *V*_TBT_ : *W*_Fe–Co50_ (1 : 1, mL : g) as the precursor material for AgNO_3_ coating. As for the TiO_2_ layer, the elemental composition of the Fe–Co50@TiO_2_@Ag magnetic material following the AgNO_3_ coating of Fe–Co50@TiO_2_ was detected by SEM-EDS. Using 10 g samples of Fe–Co50@TiO_2_, the coating conditions of different AgNO_3_ additions were studied. The ordinate of Fig. 4 is the molar ratio of Ag *vs.* Fe (Ag represents the Ag content of the coating material, Fe represents the amount of the coating material itself), and the abscissa is the feed mass of AgNO_3_ (0.4, 0.5, 0.6, 0.7, 0.8, 0.9, and 1 g, respectively). The molar ratio of Ag to Fe is used to measure the coating thickness of Ag on Fe–Co50@TiO_2_. It can be seen from Fig. 4 that the molar ratio of Ag *vs.* Fe increases with the addition of AgNO_3_. The coating amount of Ag on the surface of Fe–Co50@TiO_2_ shows almost a linear ratio with an increase in AgNO_3_ feeding amount.

**Fig. 5:**
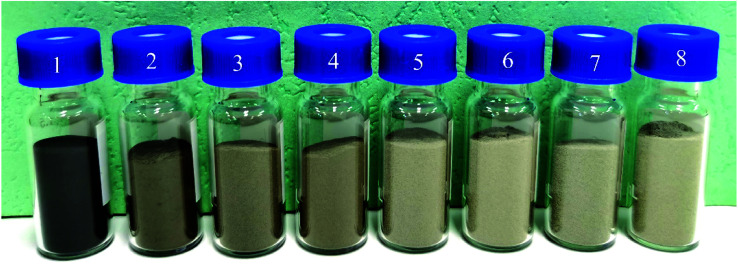
Comparison of the Ag coating amount and the color of Fe–Co50@TiO_2_@Ag. No. 1 is Fe–Co50@TiO_2_ before coating, and no 2–8 correspond to the following ratios of AgNO_3_*vs.* Fe–Co50@TiO_2_, *W*_AgNO_3__ : *W*_Fe–Co50@TiO_2__ = 0.4 : 10, 0.5 : 10, 0.6 : 10, 0.7 : 10, 0.8 : 10, 0.9 : 10, and 1 : 10, respectively.

Fe–Co50@TiO_2_@Ag magnetic materials prepared with different amounts of AgNO_3_ were put into vials, and the color changes for different coating amounts are shown in Fig. 5. Fig. 5(1) is the Fe–Co50 magnetic material before coating, Fig. 5(2)–(8) are 10 g samples of Fe–Co50@TiO_2_, with coating masses of AgNO_3_ of 0.4, 0.5, 0.6, 0.7, 0.8, 0.9 and 1 g, respectively. It can be seen from Fig. 5 that when the feed ratio of AgNO_3_*vs.* Fe–Co50@TiO_2_ reaches *W*_AgNO_3__ : *W*_Fe–Co50@TiO_2__ = 0.7 : 10 (g : g), a limit is reached and despite the feed of AgNO_3_ being increased, the color of the magnetic material does not change significantly. At this point, the color of the Fe–Co50@TiO_2_@Ag magnetic material is already white or light gray. As AgNO_3_ is relatively expensive, the most cost-effective approach, which still ensures that the color of the magnetic material is light, is using a AgNO_3_ : Fe–Co50@TiO_2_ ratio of 0.8 : 10 (g : g), such as in Fig. 5(6). In this case, the coating thickness of Ag is in the range of 20–30 nm (confirmed by SEM imaging; see Section 3.3).

**Fig. 6:**
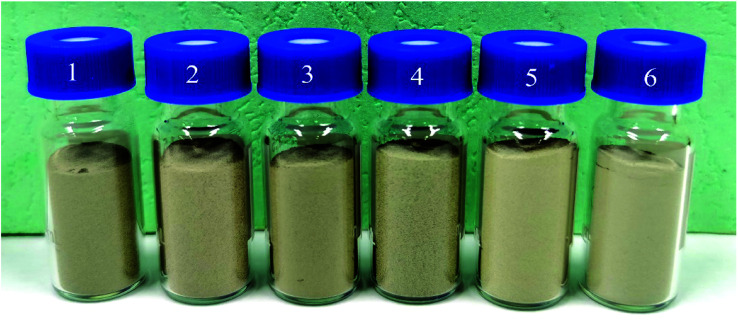
Color comparison diagram of Fe–Co50@TiO_2_@Ag with the same Ag coating amount and different TiO_2_ coating thicknesses (the feed ratio of TBT *vs.* Fe–Co50 is *V*_TBT_ : *W*_Fe–Co50_ = 1 : 3, 1.5 : 3, 2 : 3, 2.5 : 3, 3 : 3, and 3.5 : 3).

The previous experiment showed that the color of Fe–Co50 magnetic material starts to change with increasing TiO_2_ coating amount, but after reaching a limiting coating amount, the color change is not obvious. When the coating thickness of Ag reaches a certain limit, the change in color of Fe–Co50@TiO_2_@Ag magnetic material also plateaus. In order to save costs and avoid reducing the magnetic properties of these materials, this study aims to determine the optimal coating amount of TiO_2_. In this experiment, when the feeding ratio of AgNO_3_*vs.* Fe–Co50@TiO_2_ was selected as *W*_AgNO_3__ : *W*_Fe–Co50@TiO_2__ = 0.8 : 10 (g : g), the color comparison chart of Fe–Co50@TiO_2_@Ag under different TiO_2_ coating thicknesses was studied (see Fig. 6). Fig. 6(1)–(6) show the results when the feed ratios of TBT *vs.* Fe–Co50, *V*_TBT_ : *W*_Fe–Co50_, are 1 : 3, 1.5 : 3, 2 : 3, 2.5 : 3, 3 : 3, and 3.5 : 3. It can be seen from Fig. 6 that as the coating thickness of TiO_2_ increases, the color of the Fe–Co50@TiO_2_@Ag magnetic material gradually becomes lighter. When the ratio of TBT *vs.* Fe–Co50 exceeds *V*_TBT_ : *W*_Fe–Co50_ = 1 : 1 (g : g), such as in Fig. 6(5), the color of the magnetic material reaches silver-gray, and increasing the thickness of the TiO_2_ coating no longer has a significant color change. It will only result in an increase in production cost and a reduction in the magnetic properties of the material.

### 3.3 SEM analysis of magnetic materials

**Fig. 7:**
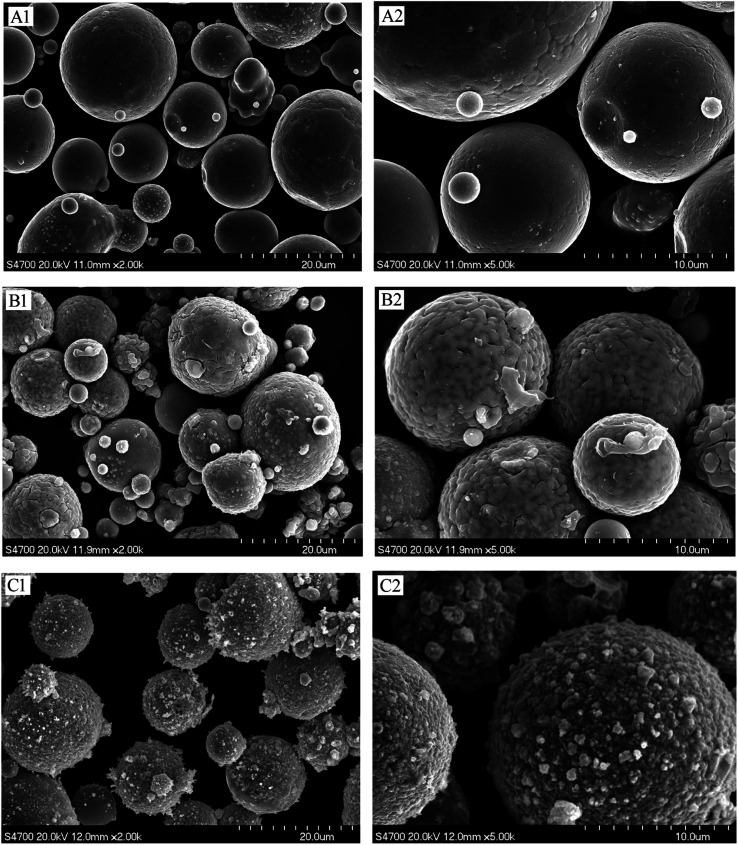
SEM pictures of Fe–Co50 before and after coating; (A1 and A2) Fe–Co50, (B1 and B2) Fe–Co50@TiO_2,_ and (C1 and C2) Fe–Co50@TiO_2_@Ag.

SEM can directly observe the surface structure and morphology of a given material, and was used to study the surface coating of the magnetic material. The surface morphologies of Fe–Co50, Fe–Co50@TiO_2_, and Fe–Co50@TiO_2_@Ag were analyzed by SEM. Fig. 7(A1 and A2) presents SEM images of the Fe–Co50 magnetic material under different magnifications. It can be seen that the material is spherical and has good dispersibility. The average particle size is greater than 10 μm, with a relatively smooth surface. Fig. 7(B1 and B2) show the surface morphology of Fe–Co50@TiO_2_. The feed ratio of TBT *vs.* Fe–Co50 is *V*_TBT_ : *W*_Fe–Co50_ = 1 : 1 (g : g); the SEM image shows that the TiO_2_ coating on the surface of Fe–Co50@TiO_2_ is complete, dense, and relatively uniform. From the cracks in the figure, it can be seen that the coating thickness of TiO_2_ is in the range of 20–30 nm. Fig. 7(C1 and C2) show the SEM surface morphology of Fe–Co50@TiO_2_@Ag. The feed ratio of AgNO_3_*vs.* Fe–Co50@TiO_2_ is *W*_AgNO_3__ : *W*_Fe–Co50@TiO_2__ = 0.8 : 10 (g : g). It can be seen from the Fe–Co50@TiO_2_@Ag SEM image that the Ag particles are densely coated on the surface of Fe–Co50@TiO_2_ with a small particle size. The particles are tightly and completely coated on the surface of the magnetic particles. The thickness is also in the range of 20–30 nm.

### 3.4 SEM-EDS analysis of magnetic materials

**Fig. 8:**
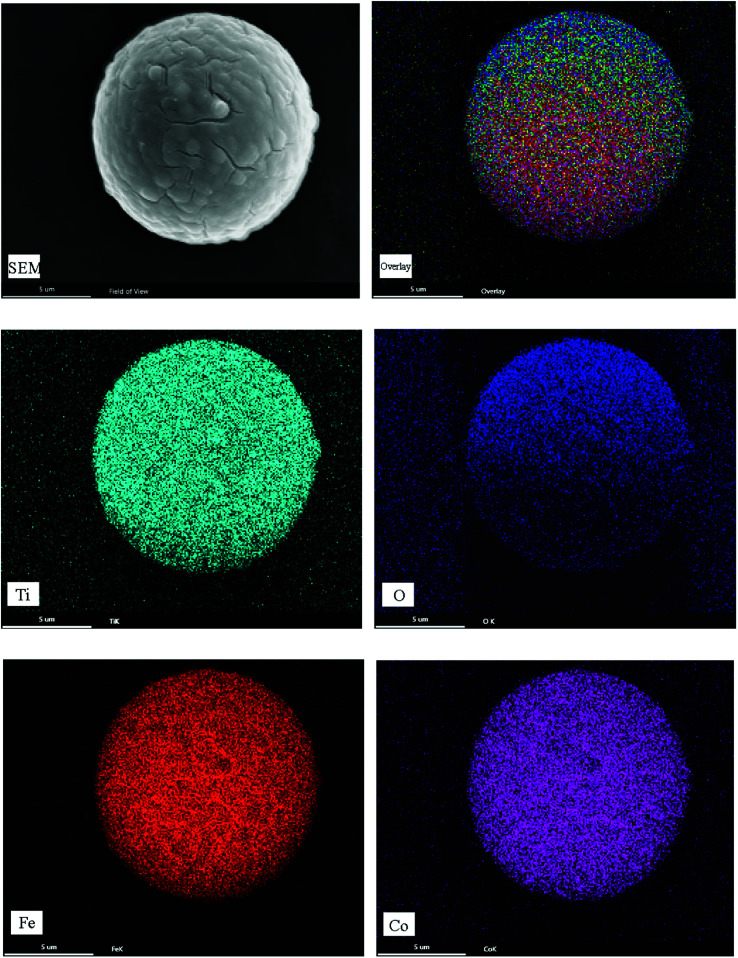
SEM-EDS mapping diagram of Fe–Co50@TiO_2_.

SEM-EDS can be used to perform qualitative and semi-quantitative analysis of the elemental composition of a material, and the distribution of the elements can be determined through surface distribution mapping. Fig. 8 is the surface distribution diagram of the prepared Fe–Co50@TiO_2_ magnetic material under the condition of *V*_TBT_ : *W*_Fe–Co50_ = 1 : 1 (mL : g). It can be seen from the SEM-EDS mapping of Fe–Co50@TiO_2_ that TiO_2_ coats the Fe–Co50 surface uniformly.

**Fig. 9:**
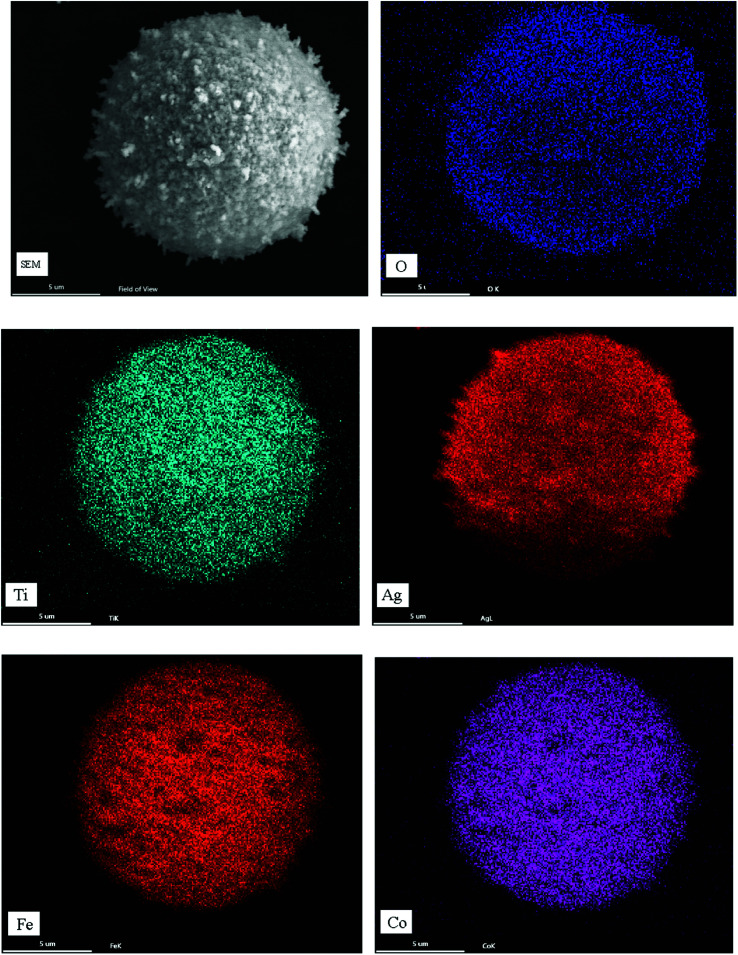
SEM-EDS element surface distribution diagram of Fe–Co50@TiO_2_@Ag.

Fig. 9 is the element distribution mapping diagram of the Fe–Co50@TiO_2_ @Ag magnetic material prepared under a feed ratio of silver nitrate *vs.* Fe–Co50@TiO_2_ of *W*_AgNO_3__ : *W*_Fe–Co50@TiO_2__ = 0.8 : 10 (g : g). The SEM-EDS surface distribution mapping clearly shows that Ag uniformly coats the surface of Fe–Co50@TiO_2_.

### 3.5 XRD analysis of magnetic materials

**Fig. 10:**
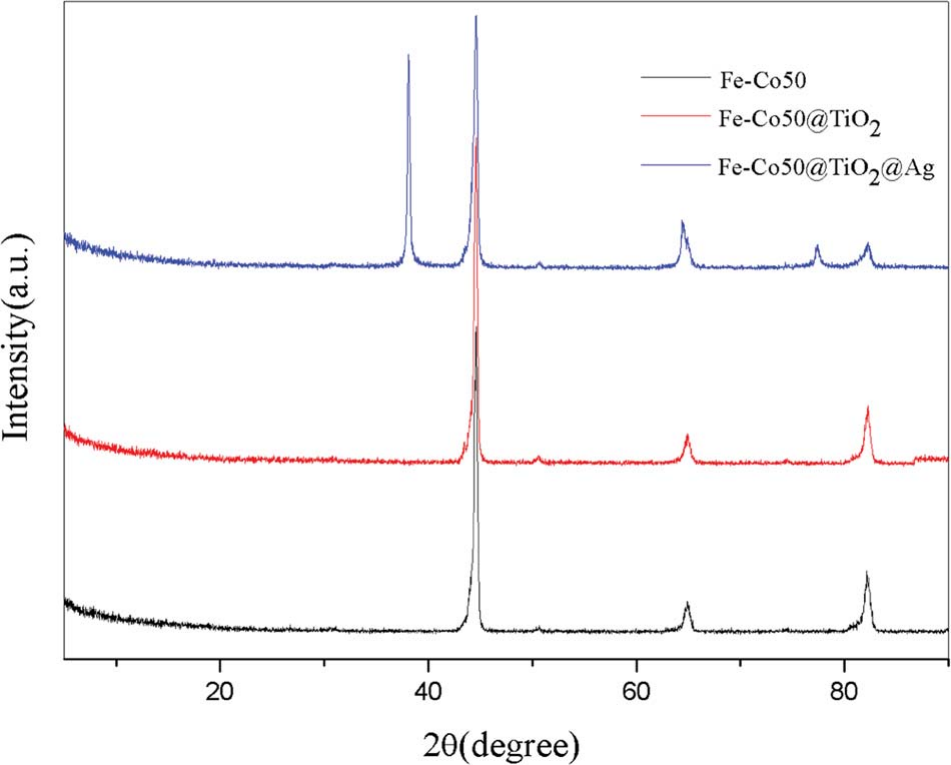
XRD diffraction patterns of Fe–Co50, Fe–Co50@TiO_2_ and Fe–Co50@TiO_2_@Ag.

Fig. 10 shows the XRD pattern before and after the magnetic material was coated. The black, red, and blue curves correspond to the X-ray diffraction results of Fe–Co50, Fe–Co50@TiO_2_, and Fe–Co50@TiO_2_@Ag, respectively. There is no diffraction peak in the Fe–Co50@TiO_2_ X-ray diffraction curve, indicating that the particle size of TiO_2_ is very small, maybe less than 2 nm, and no larger particles are formed during the process of coating Fe–Co50 with TiO_2_. This means that the Fe–Co50 surface can be evenly coated with TiO_2_ particles. The red X-ray diffraction curve of Fe–Co50@TiO_2_@Ag shows obvious metal Ag diffraction peaks, indicating that the Ag particles are relatively large. From C1 and C2 in Fig. 7, it can also be seen that the size of some Ag particles is closer to 5 nm or more.

### 3.6 Magnetic changes of the materials

**Fig. 11:**
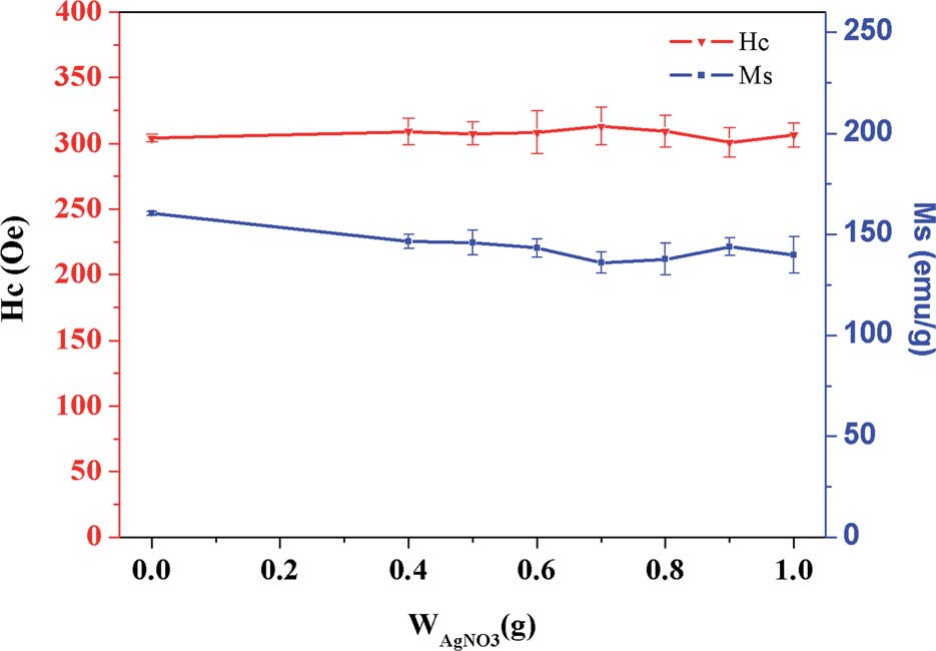
Magnetic change diagram for the same coating amount of TiO_2_ and different coating amounts of Ag. The first point corresponds to the raw material, Fe–Co50, and the second to the eighth correspond to the AgNO_3_*vs.* Fe–Co50@TiO_2_ ratios, *W*_AgNO3_ : *W*_Fe–Co50@TiO2_, of 0.4 : 10, 0.5 : 10, 0.6 : 10, 0.7 : 10, 0.8 : 10, 0.9 : 10 and 1 : 10 (g : g).

Magnetic materials are remarkable functional materials. The coercivity and remanence of these materials are the basis for their wide application. Therefore, changing the color of a magnetic material must not affect its own magnetic properties. In this study, after making the color of the magnetic material lighter by surface coating, the influence of different coating thicknesses on the weakening of magnetic properties is studied. The feed ratio of TBT to Fe–Co50 was *V*_TBT_ : *W*_Fe–Co50_ = 1 : 1 (mL : g) for the preparation of the Fe–Co50@TiO_2_ precursor. Further, the feed ratio of AgNO_3_*vs.* Fe–Co50@TiO_2_, *W*_AgNO_3__ : *W*_Fe–Co50@TiO_2__, was 0.4 : 10, 0.5 : 10, 0.6 : 10, 0.7 : 10, 0.8 : 10, 0.9 : 10, and 1 : 10 (g : g). Fig. 11 shows the measured coercivity (*H*_c_) and the saturation value of magnetization (*M*_s_) of the Fe–Co50@TiO_2_@Ag materials with different coating thicknesses by using a 7400 series vibrating sample magnetometer. With increasing coating thickness, the magnetic performance decreased slightly, but due to the reasonable control over the coating thickness in the experimental design, the performance of the magnetic material did not decrease significantly.

## 4. Conclusion

The surface of magnetic spherical particles was coated by an improved heterogeneous precipitation method. The surface of the magnetic spherical particles was first coated with a layer of TiO_2_ with excellent light-shielding qualities, followed by a layer Ag of the same excellent light-shielding ability. The Ag particles formed a core–shell structure with complete surface coating. Upon appropriately adjusting the coating thickness, the color of the coated magnetic particles became lighter without the magnetic properties of the coated magnetic particles significantly deteriorating. By adjusting the feed ratio, it was found that the optimal feed ratio of TBT *vs.* Fe–Co50 was 1 : 1 (mL : g), and the optimal feed ratio of AgNO_3_*vs.* Fe–Co50@TiO_2_ was 0.8 : 10 (g : g). After the Fe–Co50 magnetic material was coated with TiO_2_ and Ag, the element surface distribution was characterized by SEM-EDS, which showed that the magnetic particles were perfectly coated by TiO_2_ and Ag. The magnetic properties of Fe–Co50@TiO_2_@Ag with different Ag coating thicknesses were tested, and it was found that the magnetic properties of the material did not significantly decrease when a lighter color was achieved.

The shortest wavelength of visible light is approximately 400 nm. In order to conceal the surface color of dark magnetic materials, the light-colored coating must be of a thickness exceeding 400 nm. TiO_2_ has the best opacity, whiteness, and brightness; thus, coating the surface of magnetic materials with TiO_2_ can provide a good light-shielding effect, but the required amount of TiO_2_ coating results in a relatively thick layer. Furthermore, TiO_2_ can photodegrade organic matter. By coating the deposited layer of TiO_2_ with a second layer of Ag, which has no degrading effect on organic matter, we are able to not only reduce the degradation of organic matter by TiO_2_, but also make its color lighter. This work offers a solution to the difficult problem of covering the surface of magnetic particles completely, presents a method for preparing light-colored magnetic materials that broadens the application range of magnetic materials, and has high application value. Furthermore, it provides a new preparation method for the complete coating of the surface of a granular material and the formation of a core–shell structure with a complete surface.

## Conflicts of interest

There are no conflicts to declare.

## Supplementary Material
